# The Surface Roughness of Contemporary Indirect CAD/CAM Restorative Materials That Are Glazed and Chair-Side-Finished/Polished

**DOI:** 10.3390/ma17050997

**Published:** 2024-02-21

**Authors:** Ragad Albani, Syed Rashid Habib, Abdulaziz AlQahtani, Abdulaziz A. AlHelal, Mohammed Alrabiah, Saqib Anwar

**Affiliations:** 1 Department of Prosthetic Dental Sciences, College of Dentistry, King Saud University, Riyadh 11545, Saudi Arabia; 441204631@student.ksu.edu.sa (R.A.); absalqahtani@ksu.edu.sa (A.A.); abalhelal@kau.edu.sa (A.A.A.); mohalrabiah@ksu.edu.sa (M.A.); 2 Industrial Engineering Department, College of Engineering, King Saud University, Riyadh 11545, Saudi Arabia; sanwar@ksu.edu.sa

**Keywords:** surface roughness, CAD/CAM, indirect restorative materials, finishing and polishing, glazing, dental ceramics

## Abstract

The surface roughness (Ra) of indirect computer-aided design/computer-aided manufacturing (CAD/CAM)-fabricated dental restorations is crucial for their long-term durability. This study intended to evaluate the Ra of five different types of contemporary indirect CAD/CAM restorative materials with varying compositions that were glazed and finished/polished. A total of 75 specimens, disc-shaped (10 mm × 2 mm), were obtained from five materials (*n* = 15) (Tetric CAD, IPS e.max CAD, IPS e.max ZirCAD, CELTRA Duo, and Vita Enamic) and fabricated by CAD/CAM. One of the two surfaces for each specimen was subjected to glazing, while the other surface was subjected to finishing/polishing. The Ra of the two surfaces in micrometers (μm) was evaluated using a Profilometer, while the surface topography was examined using a scanning electron microscope. Using SPSS, the Kruskal–Wallis, post hoc Conover, and Mann–Whitney tests were used to statistically evaluate the data. A comparison of the Ra for the finished/polished surfaces of the five test materials showed significant differences (*p* < 0.0001). Among the finished/polished surfaces, the mean rank values of Vita Enamic were significantly higher than the other four test materials (*p* < 0.0001). A comparison of the Ra of glazed surfaces among the five study materials revealed significant differences (*p* < 0.0001). The Ra for the IPS e.max ZirCAD material was significantly higher than the rest of the four materials (*p* < 0.001). A comparison of the Ra for two types of surface conditioning within each of the five test materials showed a significant difference (*p* < 0.05). Only for IPS e.max ZirCAD was the Ra of the glazed surface significantly higher than the finished/polished surface (*p* < 0.0001). Significant variations in the surface roughness (Ra) were exhibited between the finished/polished and glazed surfaces of the five test materials. Hybrid ceramics showed the highest Ra values for the finished/polished surfaces, and zirconia exhibited the highest Ra values among the glazed surfaces among the tested materials. The Ra values of either finished/polished or glazed surfaces of the test materials were within the clinically acceptable range (0.2–0.5 μm), except for the glazed surface of the zirconia ceramics (0.84 μm).

## 1. Introduction

The use of indirect restorative materials in computer-aided design and computer-aided manufacturing (CAD/CAM) is growing in accessibility and ease. With the use of this technology, dentists are able to design and deliver many types of prostheses in a more efficient way [1]. The CAD/CAM can be either subtractive (milling) or additive (three-dimensional printing). For ceramics, various types of prefabricated blocks and discs are available on the market that can be designed and milled using CAD/CAM [2]. However, material wastage and high cost are considered disadvantages of this technology. Modern restorative materials for CAD/CAM are designed with superior mechanical and clinical properties, which ensure the long-term success of these restorations [3].

To produce a successful prosthetic restoration, the technique must always be customized to each individual patient. In order to meet the patient’s individual needs, the selection of the material and method must be individualized for each case. As manufacturers strive to develop a dental material that fulfills the ideal biological, mechanical, as well as aesthetic requirements, metal-free restorative materials have been developed [4]. These materials have excellent optical properties and physio-mechanical properties, including high strength, biocompatibility, wear resistance, and good polishability. For previously mentioned reasons, metal-free restorative materials are considered the main treatment option for indirect restorations, such as crowns, veneers, inlays, and onlays [5].

Kelly and Benetti have proposed that ceramics can be categorized into three primary groups according to their composition: (1) glass-dominated, (2) particle-filled glass, and (3) polycrystalline [6]. Predominantly, glass in the form of feldspathic glass–ceramic consists of amorphous alumino-silicate glass made from feldspar crystals and has the best aesthetic matching to natural teeth. However, it has very low strength, limiting its use in veneering materials to either metal or all-ceramic frameworks [6,7]. In particle-filled glass, different types of fillers have been incorporated to improve the mechanical properties of feldspathic porcelain. Lithium disilicate in chair-side CAD/CAM blocks is an example of these fillers [8]. Others have incorporated zirconia (Zr), leading to further improvement in the mechanical characteristics of these materials [9]. On the other hand, polycrystalline ceramics are densely packed crystals of alumina or Zr with no glassy matrix, which will have a low translucency and cannot be etched [6]. Recently, polymer-infiltrated resin ceramics have been developed, which exhibit less wear to their antagonists and have physical properties superior to conventional composites [10].

One of the most crucial variables to take into account for the lifetime and efficacy of the indirect restorations that patients receive is surface roughness (Ra). It is recommended to have an Ra value between 0.2 um and 0.5 um to ensure the best prosthetic performance [11,12]. In daily clinical practice, indirect restoration often requires some modification and adjustment before or after cementation in order to correct contour and/or occlusion. This modification results in the loss of surface glazes and a rough surface, leading to more wear of the antagonist teeth and development of microcracks, which can propagate and cause prosthesis fracture [13,14]. A smooth restoration surface should be ensured to avoid such complications and to increase the longevity of the provided treatment [15,16].

In the literature, there is controversy regarding the best method used to achieve the smoothest surface of the indirect restorations. Some studies have found that glazing is superior to mechanical polishing [17,18]. Others have shown comparable results between the two methods [11,16]. Others have concluded that mechanical polishing is superior to glazing in terms of smoothness [19]. Ideally, the altered prosthetic surface should be subjected to repolishing and glazing prior to final prosthetic cementation. However, reglazing requires several appointments as it is performed in the laboratory. Thus, chair-side polishing is faster and easier, and different kits are available on the market, including sandpaper discs, fine diamond burs and paste, rubber cups, and wheels [20,21,22]. Inconsistent results are found regarding the standardized and recommended chair-side finishing/polishing systems due to variations in the materials used, methods of evaluation, and different measuring parameters [23,24,25].

As mentioned previously, numerous restorative materials with diverse compositions are available on the market due to advancements in the field of dental material sciences. Additionally, the makers are attempting to blend components in an effort to combine their benefits and improve the materials’ lifespan and clinical performance relative to their original composition. Nonetheless, there are not many studies in the literature that have examined the surface characteristics of these recently created materials. Thus, this study’s objective was to assess and compare the Ra values of glazed and chair-side-finished/polished surfaces of five commonly used contemporary indirect CAD/CAM restorative materials (Tetric^®^ CAD, IPS e.max CAD, IPS e.max ZirCAD, CELTRA Duo, and Vita Enamic) by using a Profilometer. In addition, the surface topography of the glazed and polished surfaces of the tested materials was also evaluated by using a scanning electron microscope (SEM).

## 2. Materials and Methods

Study Setting:

This study was conducted at King Saud University’s Industrial Engineering Laboratory and the College of Dentistry Research Center (CDRC), located in Riyadh, Saudi Arabia.

Ethical Approval:

An ethical approval was obtained from the CDRC (No. PR 0143) and the Ethical Committee of the Institutional Review Board (IRB) (No. E-22-7201) of King Saud University Medical City (KSUMC), Riyadh.

Study Materials:

Five types of the latest and most advanced indirect CAD/CAM restorative materials with varying compositions and unique properties were investigated and tested for their surface roughness and topography. The details of the materials are presented in Table 1.

Sample Size Calculation:

The power analysis was carried out using G*Power 3.1.9.3 freeware analysis software (G*Power, Heinrich-Heine-Universität Düsseldorf, Germany) for sample size calculation. With an alpha of 0.05, a power of 0.85 (85%), and a medium effect size of 0.5, the total sample size calculated was 75 which was obtained from five materials (*n* = 15).

**Figure 1:**
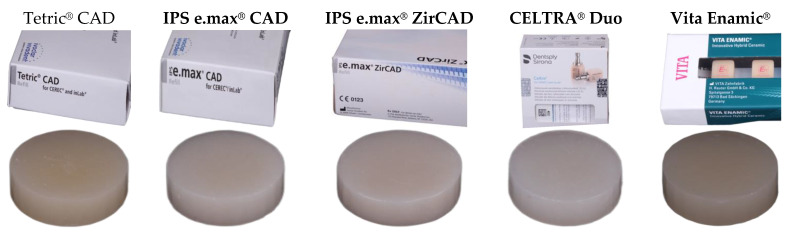
Test materials with their corresponding specimens (10 mm diameter and 3 mm thickness).

Specimens Preparation:

Seventy-five-disc shape specimens of a 10 mm diameter and 3 mm thickness were designed using ExoCad software (https://exocad.com/) and milled with Ceramill motion 2 milling machine. Following that, crystallization was carried out according to the manufacturer’s instructions for Tetric^®^ CAD; IPS e.max^®^ CAD; CELTRA^®^ Duo; and Vita Enamic^®^, whereas IPS e.max^®^ ZirCAD was sintered. Finally, glazing was carried out as per the recommendations of the manufacturers for all the specimens to one surface only. Material brands and their final specimens after milling are shown in Figure 1.

**Figure 2:**
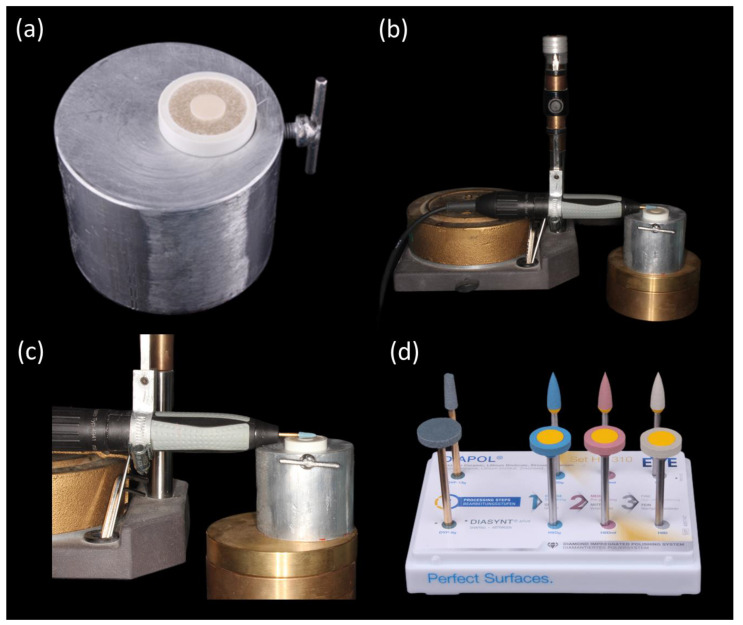
(**a**) Specimens mounted in a mold, (**b**) mounted hand piece in relation to the specimens, (**c**) close up view and (**d**) finishing and polishing kit.

Finishing and Polishing Protocol:

For finishing, a diamond-impregnated system (DIASYNT^®^Plus; EVE Ernst Vetter GmbH, Neureutstr. 6, 75210 Keltern, Germany) was used as it has a high abrasive rate with minimal heat resistance without the need of a water-cooling system. For polishing, a three steps diamond-impregnated polishing system (DIAPOL^®^ Set HP 310; EVE Ernst Vetter GmbH, Neureutstr. 6, 75210 Keltern, Germany) was utilized. It consisted of coarse, medium, and fine grits which provided a smooth surface without the need of additional glazing, and can be used for variety of ceramic materials [26,27]. For simulating the clinical scenario and standardization, the following protocol was adopted uniformly for all the tested specimens:

1. All the specimens were mounted in a mold as shown in Figure 2a.
2. A handpiece was mounted in a dental surveyor in order to control the movement and strokes of the burs to the surfaces of the specimens (Figure 2b,c).
3. Finishing was performed using a diamond-impregnated system (DIASYNT^®^Plus; EVE Ernst Vetter GmbH, Neureutstr. 6, 75210 Keltern, Germany) in one direction for 60 s to the full surface (Figure 2d).
4. Smoothening was performed using DIAPOL^®^ Set HP 310 (EVE Ernst Vetter GmbH, Neureutstr. 6, 75210 Keltern, Germany) coarse grit in one direction for 60 s to the full surface (Figure 2d).
5. Pre-polishing was performed using DIAPOL^®^ Set HP 310 (EVE Ernst Vetter GmbH, Neureutstr. 6, 75210 Keltern, Germany) medium grit in one direction for 60 s to the full surface (Figure 2d).
6. Lastly, high-shine polishing was performed using DIAPOL^®^ Set HP 310 (EVE Ernst Vetter GmbH, Neureutstr. 6, 75210 Keltern, Germany) fine grit in one direction for 60 s to the full surface (Figure 2d).

**Figure 3:**
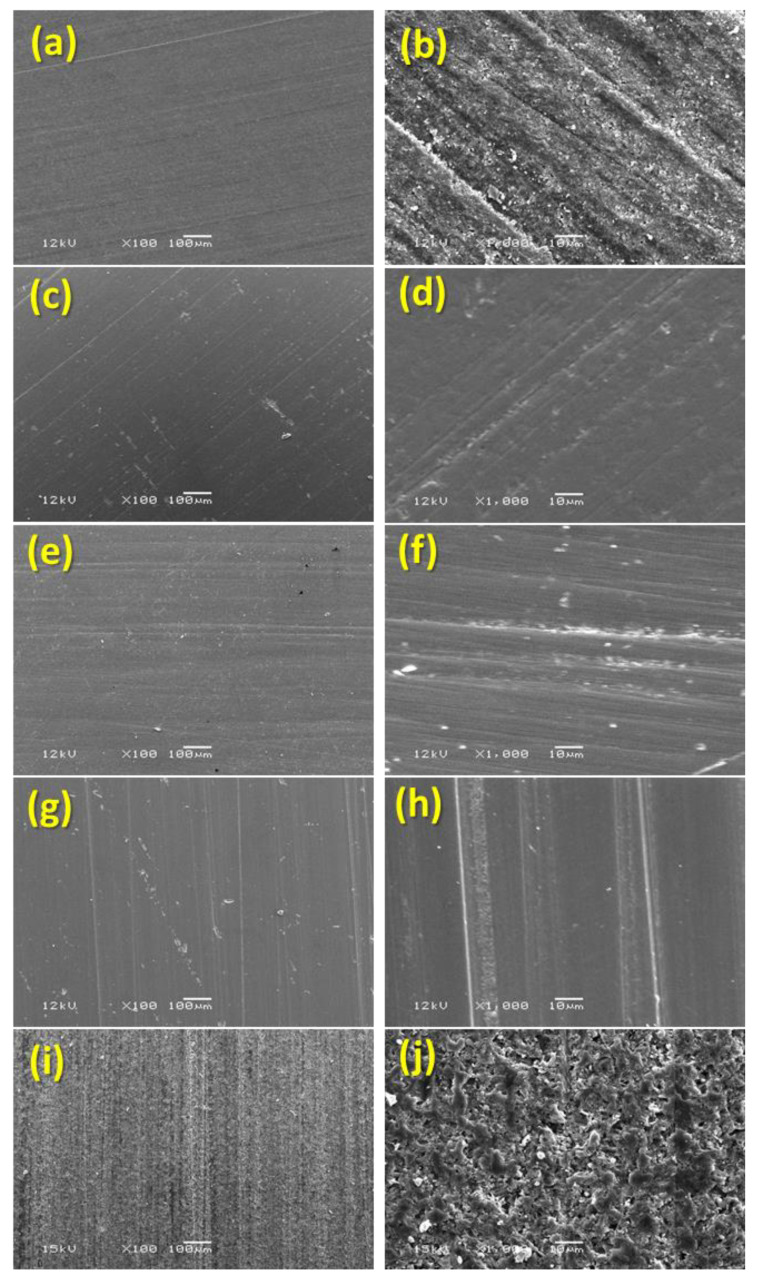
SEM images on finished/polished surface. (**a**): Tetric^®^ CAD 100×; (**b**): Tetric^®^ CAD 1000×; (**c**): IPS e.max CAD 100×; (**d**): IPS e.max CAD 1000×; (**e**): IPS e.max ZirCAD 100×; (**f**): IPS e.max ZirCAD 1000×; (**g**): CELTRA Duo 100×; (**h**): CELTRA Duo 1000×; (**i**): Vita Enamic 100×; (**j**): Vita Enamic 1000×.

**Figure 4:**
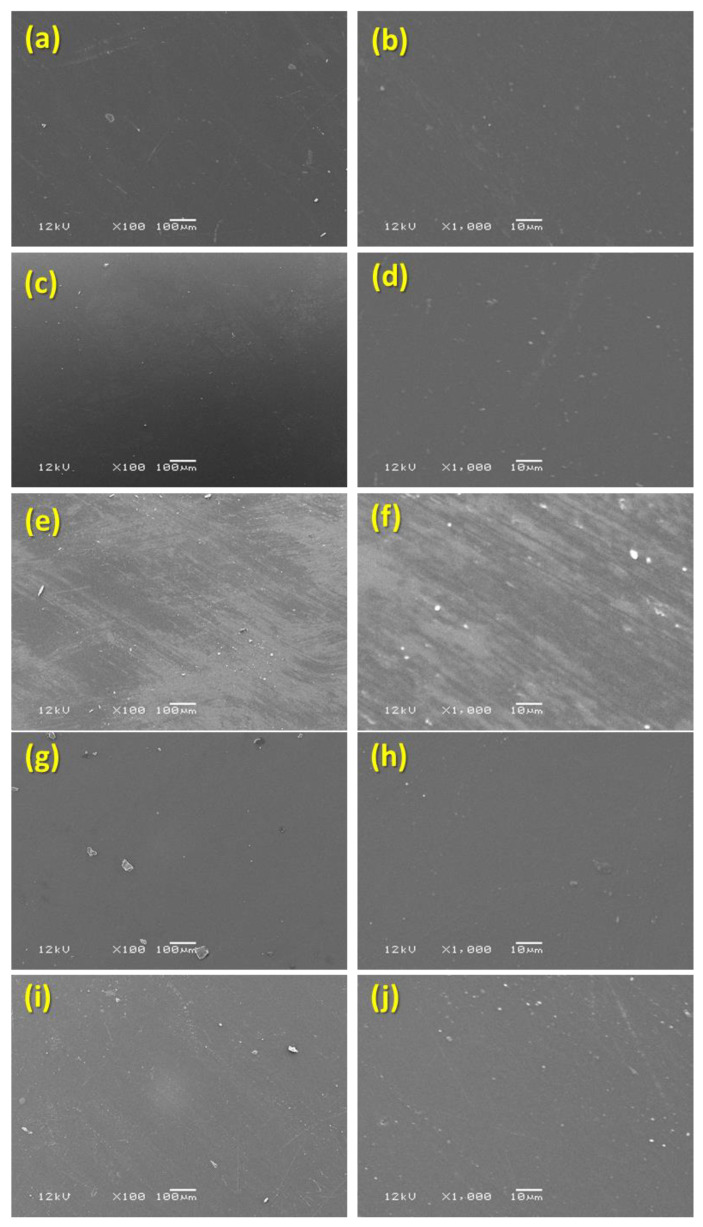
SEM images of glazed surface. (**a**): Tetric^®^ CAD 100×; (**b**): Tetric^®^ CAD 1000×; (**c**): IPS e.max CAD 100×; (**d**): IPS e.max CAD 1000×; (**e**): IPS e.max ZirCAD 100×; (**f**): IPS e.max ZirCAD 1000×; (**g**): CELTRA Duo 100×; (**h**): CELTRA Duo 1000×; (**i**): Vita Enamic 100×; (**j**): Vita Enamic 1000×.

Scanning Electron Microscope (SEM) Analysis:

Prepared specimens with the two types of surface conditioning, i.e., finished/polished surfaces and glazed surfaces were examined and photographed for qualitative assessment using SEM (EVO-LS10; Scanning Electron Microscope; Carl Zeiss Microscopy GmbH, Jena, Germany) after being coated with gold sputtering. Random images were recorded for each specimen at different magnifications (100× to 1000×) as shown in Figure 3 (finished/polished surface) and Figure 4 (glazed surface).

Data Analysis:

IBM SPSS Statistical software for Windows version 26.0 (IBM Corp., Armonk, NY, USA) was used to analyze the data. The Ra levels were described using descriptive statistics (mean, standard deviation, median, and interquartile range). As the Ra values were not following a normal distribution, the non-parametric statistical tests: (i) the Kruskal–Wallis test followed by post hoc Conover test were used to compare the mean rank values of Ra among the five test materials (Tetric^®^ CAD; IPS e.max^®^ CAD; IPS e.max^®^ ZirCAD; Celtra^®^ Duo; Vita Enamic) in each of the surfaces (finished/polished and glazed); (ii) the Mann–Whitney U-test was used to compare the mean rank values of Ra between two surfaces (finished/polished and glazed) within each of the five test materials. A *p*-value of less than 0.05 was considered to report the statistical significance of results.

## 3. Results

The descriptive statistics (mean, standard deviation, median, and interquartile range) of Ra for each of the five test materials in relation to the two types of surfaces (finished/polished and glazed) are presented in Table 2.

The comparison of mean ranks of Ra of the finished/polished surfaces for the five study materials showed high statistically significant difference in the Ra values (*p* < 0.0001). The post hoc test showed that the mean rank values of IPS e.max ZirCAD material was significantly higher than the other three materials (Celtra Due, IPS e.max CAD, and Tetric CAD) (*p* < 0.001) and significantly lower than the Vita Enamic material (*p* < 0.001). Also, the mean rank values of the Vita Enamic material were significantly higher than all the other four materials (Celtra Due, IPS e.max CAD, Tetric CAD, and IPS e.max ZirCAD) (*p* < 0.0001). However, there was no statistically significant difference in the mean rank values across the three materials (Celtra Due, IPS E max, and Tetric CAD) (*p* > 0.05) (Table 3). The Vita Enamic showed the roughest surfaces out of all the five test materials, which was clearly apparent and supported by the results of the surface analysis of the SEM images (Figure 3j). Lower-hardness resin is easily removed from the hybrid group ceramics due to their porous nature, and this phenomenon may have led to a notably higher Ra. Resin matrices may have contributed to some of the porous surfaces shown in the composite material’s SEM pictures (Figure 3b).

The comparison of mean ranks of Ra among five study materials (Tetric CAD, IPS e.max CAD, IPS e.max ZirCAD, Celtra Duo, and Vita Enamic) by using a glazed Ra surface showed a high statistically significant difference in the Ra values (*p* < 0.0001). The post hoc test showed that the mean rank values of the IPS e.max CAD material was significantly higher than the two materials (Celtra Due and Tetric CAD) (*p* < 0.001) but significantly lower than the mean rank values of the IPS e.max ZirCAD material (*p* < 0.001) and not significantly different from the mean rank values of the Vita Enamic material (*p* > 0.05). Also, the mean rank values of the IPS e.max ZirCAD material was significantly higher than all the four materials (Celtra Due, IPS e.max CAD, Tetric CAD, IPS e.max ZirCAD, and Vita Enamic) (*p* < 0.001). However, there was no statistically significant difference in the mean rank values across the three materials (Celtra Due, Tetric CAD, and Vita Enamic) (*p* > 0.05) (Table 4). The IPS e.max ZirCAD demonstrated the roughest surfaces out of all the five test materials, which was also obvious and supported by the findings from the surface analysis of the SEM pictures (Figure 4e,f).

**Figure 5:**
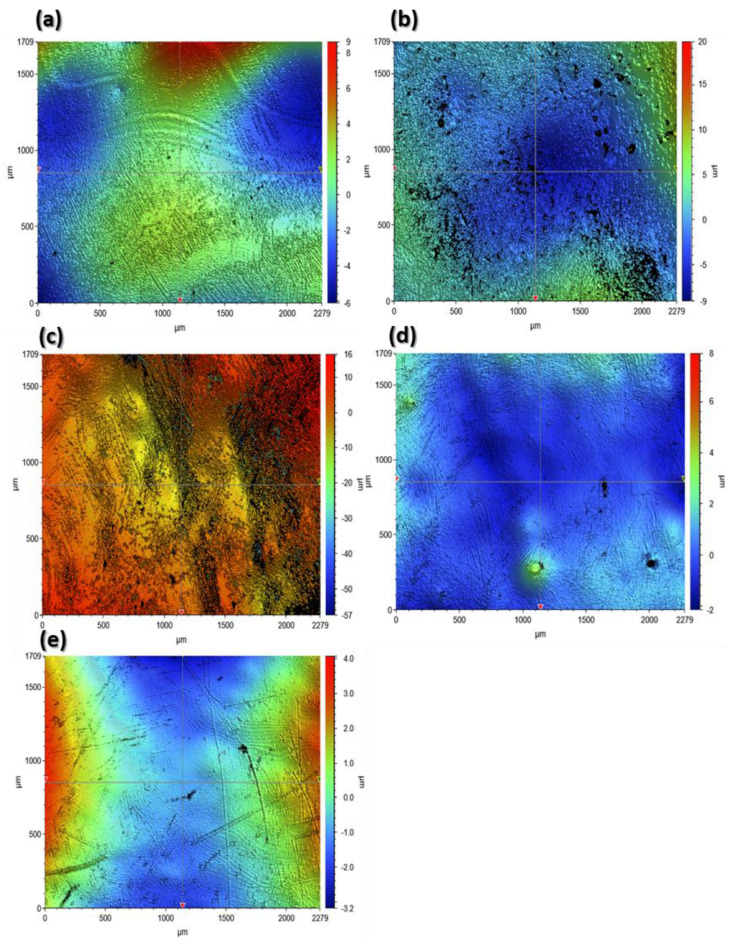
The 3D profilometric contours of the scanned surface on the glazed specimens. (**a**): Tetric^®^ CAD; (**b**): IPS e.max, CAD; (**c**): IPS e.max ZirCAD; (**d**): CELTRA Duo; (**e**): Vita Enamic.

**Figure 6:**
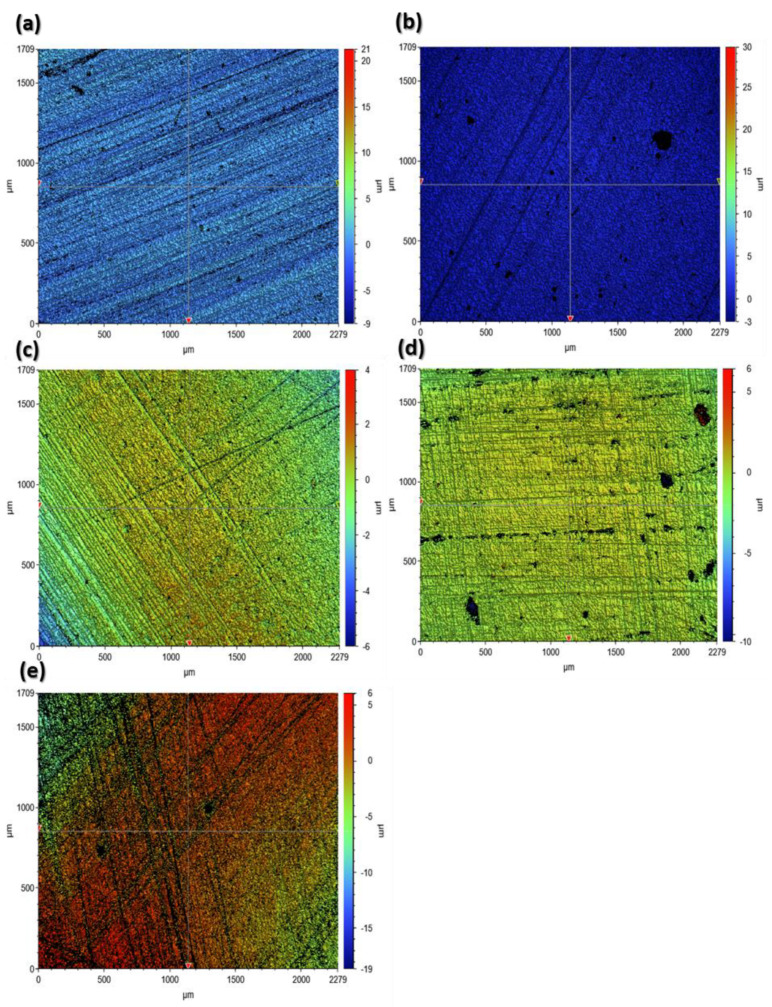
The 3D profilometric contours of the scanned surface on the polished specimens. (**a**): Tetric^®^ CAD; (**b**): IPS e.max; CAD (**c**): IPS e.max ZirCAD; (**d**): CELTRA Duo; (**e**): Vita Enamic.

Figure 5 and Figure 6 present sample profilometeric images on glazed and finished surfaces of the tested specimens. The glazed specimens showed a smoothed topography, while all the polished specimens showed the scratch marks of the diamond burr used for polishing. Among the glazed specimens, the IPS e.max ZirCAD (Figure 5c) showed an abnormally higher peak to valley range from −57 µm to 16 µm. However, by carefully observing, it can be noticed that majority of the deeper points (represented by a dark blue color) are lying within the black zones, which represent the areas where the optical profiler was unable to capture the data. Black zones are mostly created due to the surface reflectivity issues. Apart from IPS E.max ZirCAD samples (Figure 5c and Figure 6c), the polished samples always showed a higher range of peak to valley variation compared to the corresponding glazed specimens.

**Figure 7:**
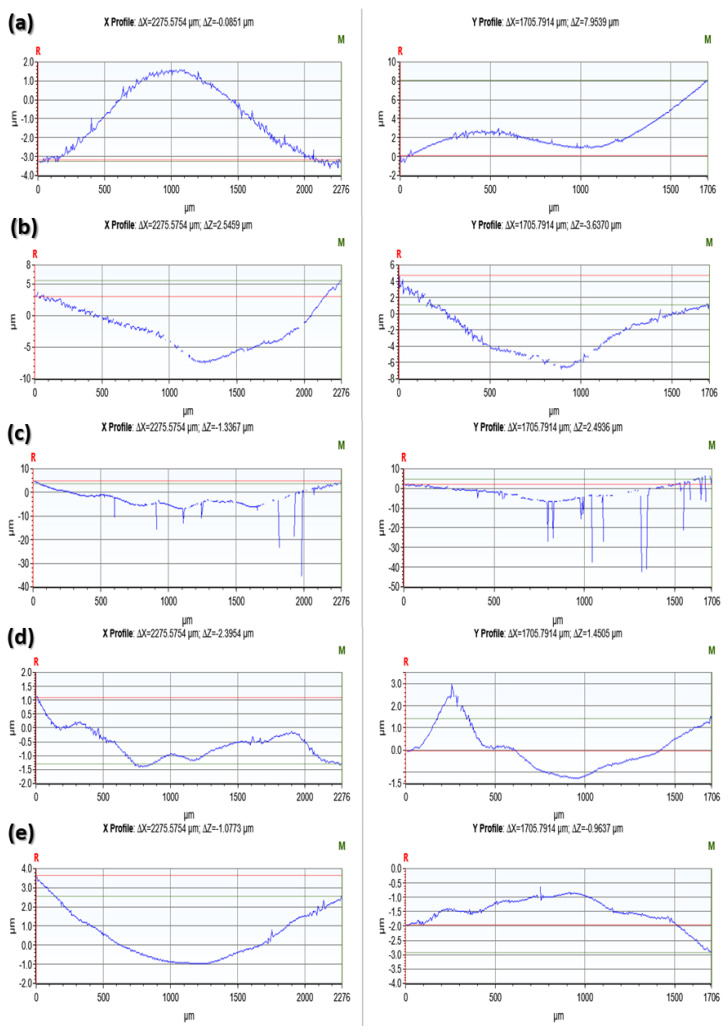
Surface roughness profiles of the glazed specimen surfaces extracted from the 3D profilometric contours shown earlier in Figure 6. (**a**): Tetric^®^ CAD; (**b**): IPS e.max, CAD; (**c**): IPS e.max ZirCAD; (**d**): CELTRA Duo; (**e**): Vita Enamic.

**Figure 8:**
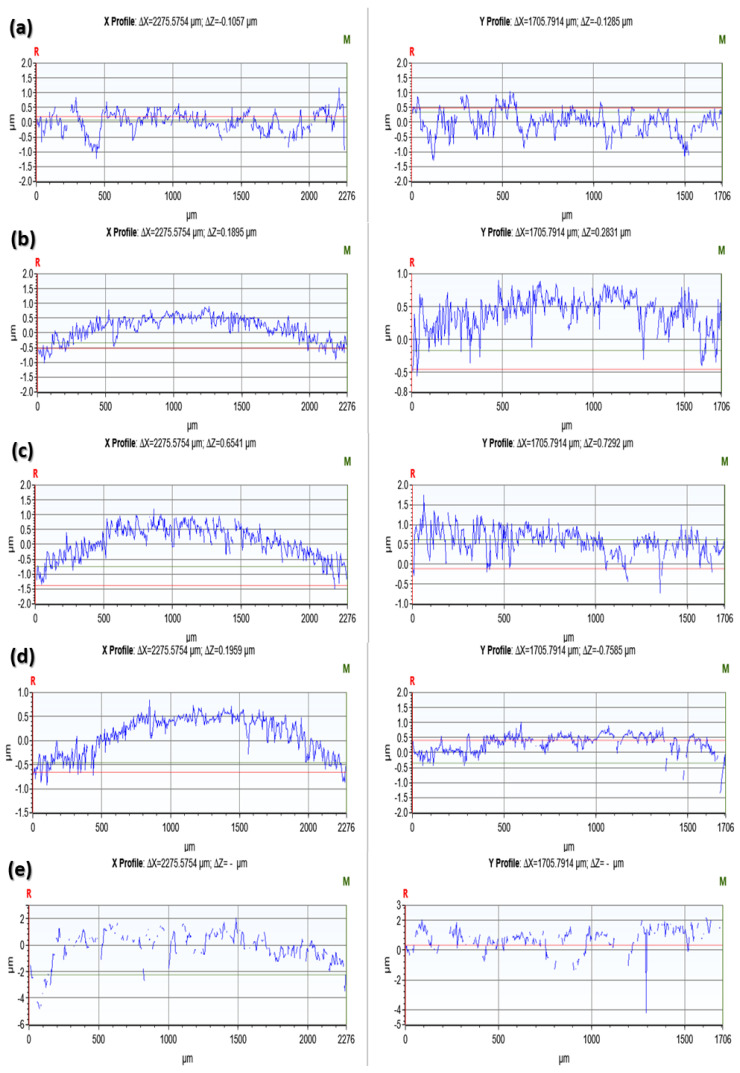
Surface roughness profiles of the polished specimen surfaces extracted from the 3D profilometric contours shown earlier in Figure 7. (**a**): Tetric^®^ CAD; (**b**): IPS e.max, CAD; (**c**): IPS e.max ZirCAD; (**d**): CELTRA Duo; (**e**): Vita Enamic.

Figure 7 and Figure 8 show the 2D roughness profiles of the specimens extracted along the horizontal (X-direction) and vertical (Y-direction) from the middle of the 3D contours. The Ra profiles for the glazed specimens are usually smoother (50% to 220%, as shown by Ra values in Table 2) compared to the corresponding roughness profiles of the polished specimens. This is because the pores that the ceramic materials inherently possess are suppressed after glazing. However, IPS e.max ZirCAD (glazed) showed higher roughness as compared to its counterpart polished surface (Figure 7c and Figure 8c). This could be because of the high noise captured in the case of the glazed specimen (Figure 7c, sudden negative/deep spikes) due to its higher surface reflectivity, as the white light optical profilometry used in this work is very sensitive to the surface optical characteristics, which are beyond the scope of the current work.

**Figure 9:**
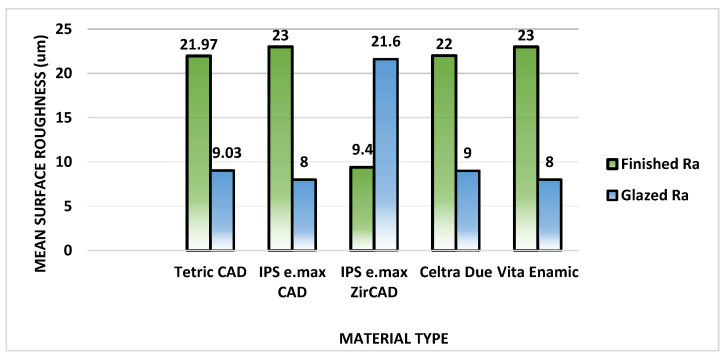
Comparison of mean surface roughness between two surfaces (finished and glazed) within each of five study materials.

The comparison of mean ranks of Ra between two types of surfaces (finished/polished and glazed) in each of the five study materials showed a high statistically significant difference in the values of Ra. For four study materials (Celtra Due, IPS e.max CAD, Tetric CAD, and Vita Enamic), the mean rank values of Ra by using the finished/polished surface are significantly higher than the mean ranks values of Ra by using glazed surface (*p* < 0.0001). Meanwhile, for the IPS e.max ZirCAD material, the mean rank values of Ra using glazed surface are significantly higher when compared with the mean rank values using finished/polished surface (*p* < 0.0001) (Table 5) (Figure 9).

## 4. Discussion

In this in vitro study, test specimens with the same shape and dimensions were used and a 3D non-contact profilometer was used to measure the surface roughness (Ra) values in micrometers (μm) of the polished and glazed surfaces of five contemporary up-to-date indirect CAD/CAM restorative materials. A scanning electron microscope (SEM) was also used to scan the surface topography of the polished and glazed surfaces of the materials that were being tested. Many scholars have stated that the approach used in this study to measure the Ra parameter using a 3D non-contact profilometer provides good resolution of the traced surface and is one of the most recommended methods to measure the Ra [28,29]. A 3D non-contact optical profilometer interference microscope, has been demonstrated to be more dependable and superior in quantitative surface topographic investigation. In order to produce an accurate qualitative representation of the sample, it is additionally attached to a camera that takes a 3D surface texture image of the entire sample [30,31]. The Ra parameter continues to be a useful general surface topography guideline, offering a practical and understandable value that permits the comparison of the Ra of various materials as well as the comparison of the findings with other studies and standards [32]. Further helpful information that is connected to the Ra values of the tested materials was also obtained by using SEM to evaluate the test specimens’ surfaces.

The Ra of five contemporary CAD/CAM dental restorative materials with various chemical compositions were investigated in the current study. The advantage of CAD/CAM blocks is their industrial production, which eliminates the possibility of processing flaws and guarantees standardization. Each of the five materials that were evaluated had a different composition, esthetics, and physical attributes [8,33]. The advantages of these unique materials over each other are claimed by their manufacturers and have been tested and reported for different properties in several research studies. The present study tried to investigate and compare the surface properties of these materials by testing the glazed and finished/polished surfaces of these materials. Ideally, all these materials should exhibit similar surface properties [34]; however, the Ra of both the glazed and finished surfaces of the tested specimens behaved differently and showed significant variations according to statistical analysis. Thus, the null hypothesis of similar Ra and no changes between the Ra for the glazed and polished/finished surfaces of these five tested indirect CAD/CAM restorative materials was rejected.

One metric used to assess the quality of the outermost surface is roughness. Higher Ra is mostly caused by flaws and textures, which can affect how well a dental material works and how long it lasts [35]. Additionally, owing to the dispersion action, it increases the opacity and decreases the translucency of zirconia. Composite (Tetric^®^ CAD), lithium disilicate glass-ceramics (IPS e.max^®^ CAD), zirconium oxide ceramics (IPS e.max^®^ ZirCAD), Zr-reinforced lithium silicate (CELTRA^®^ Duo), and hybrid ceramic (Vita Enamic^®^) were the five materials that were tested in terms of composition. There were no appreciable variations in the mean values of the Ra produced by the zirconia-reinforced lithium silicates, lithium disilicate glass ceramics, and composites with regard to the final surfaces. Zirconia and hybrid ceramics, on the other hand, displayed greater Ra values; the hybrid ceramics had the greatest Ra. For the aim of uniformity, the same investigator finished and polished each of the five examined group samples using the same process in order to obtain a smooth surface. Because of its crystalline structure and material hardness, zirconia’s high Ra seems reasonable and has been previously reported in the literature [36,37]. The rougher surface for this test group’s materials may have resulted from sintering-induced pores and grain boundary fissures, which weaken zirconia and reduce its structural endurance. Furthermore, zirconia’s higher hardness and rougher surface could encourage the wearing of opposing teeth [37,38].

The hybrid ceramics have a porous structure created by resin polymer seeping into ceramic blocks and are made up of 86% ceramic network and 14% polymer network [39,40]. Because of its porous nature, lower-hardness resin can be easily removed from the hybrid group ceramics in the current investigation due to external factors like polishing and finishing [40]. This phenomena may have resulted in noticeably higher Ra. The porous surfaces of the hybrid ceramic were clearly noticeable in the scanning electron microscopic images for hybrid ceramics as compared to the other materials. However, the images for the composite material also showed some porous surfaces; the reason could be the presence of resin matrices. The images for the rest of the three materials were almost identical showing the uniform surfaces after finishing/polishing at different magnifications from 100× to 1000×. The production of heat during the finishing and polishing of the specimens is one potential explanation for the porous surfaces of the hybrid ceramics [40,41]. The temperature rises above the glass transition point during the polishing operation because the resin composite is a poor heat conductor and retains heat in the outer layer of the material [40,42].

The process of covering porcelain surfaces with a vitreous material or impermeable coating after they have been fired and fused is known as glazing [43]. Because it can improve surface smoothness, fracture resistance, and minimize the ceramic surface’s potential abrasiveness by closing open pores, a glazed ceramic surface is typically seen as advantageous [44]. Zirconia had the highest Ra values with significant differences amongst all four tested materials when Ra levels for the materials were compared after glazing. The chemical makeup and particle size of the zirconia ceramic may be connected to these high Ra values. A total of 85–92% of ZrO_2_ makes up the zirconium oxide ceramics (IPS e.max^®^ ZirCAD) utilized in this investigation [45]. At ambient temperature, ZrO_2_ crystallizes as a monoclinic; at higher temperatures, it becomes tetragonal and cubic. Zirconia changes phases disruptively when heated. After cooling from high temperatures, the structure cracks due to significant strains induced by the volume shift brought on by the structure’s transition from tetragonal to monoclinic to cubic [46,47]. It has also been reported as utilizing a finishing and polishing procedure instead of glazing to create a more natural texture because glazing typically wears off over time [48]. When comparing zirconia to the other materials, the scanning electron microscopy images made it evident that the glazed zirconia had a rougher surface. The smooth surfaces of the remaining four materials were depicted in nearly identical photographs at varying magnifications ranging from 100× to 1000×.

Numerous investigations have indicated that the range of Ra in glazed ceramics is between 0.2 μm and 0.5 μm [43]. Numerous factors, including the types of various ceramic materials, polishing agents, operator error, human error, and profilometer calibration, could be to blame for these differences in the results [49]. Based on the available literature, 0.5 μm is deemed clinically suitable for ceramic restoration. This study’s finished/polished and glazed specimens’ surface roughness (Ra) varied between 0.18 μm and 0.46 μm and between 0.08 μm and 0.84 μm for each of the five test materials. Regardless of the specimens’ glazing or polishing, these values fell within the clinically acceptable ranges for all test materials. The threshold over the clinical acceptance was only seen on the glazed surface of the zirconia (0.84 μm) material; however, it was only 0.34 μm higher, which may not be very critical from a clinical standpoint as it is common to see differences in the Ra of chair-side polishing and glazing for restorative materials [34,43]. A literature search on the subject uncovered varying perspectives regarding the two forms of surface conditioning. In their research, Wright et al. demonstrated that chair-side polishing is just as good as or superior to glazing [50]. Only a handful of research studies have demonstrated a contrary outcome, namely, that chair-side polishing is inferior to glazing [51,52,53]. In a study using Vita VMK porcelain, Haralur SB assessed the effectiveness of the Shofu polishing kit. According to the findings, glazed surfaces had the lowest surface roughness ratings [24]. In contrast to polished surfaces, Rashid H’s study found that the glazed porcelain surfaces of VITA VMK were rougher. Nonetheless, in their research, both writers employed comparable porcelain specimens but distinct polishing methods [54]. There could be multiple explanations for the differences in findings across several research. The degree of sintering or condensation of dental porcelain particles determines the quantity and dimensions of surface pores that are opened as a result of grinding. Two samples were utilized in each study: one for chair-side polishing and the other for glazing or reglazing. As a result, it is impossible to determine if the condensation in the two samples was uniform. The specimens’ surfaces were ground using medium- or low-grit diamond points since high-grit points cause the pores to open up more while grinding. Chair-side polishing and reglazing these big pores are not easy ways of sealing them. The use of various ceramic materials for the specimens, various polishing materials, operators’ characteristics during specimen preparation and assessing surface roughness could all be responsible for variations in the results [55].

There were limitations with this investigation. The manufacturing and fabrication protocols of each material varied, which may have affected the surface behavior. Furthermore, for the uniformity and standardization, the authors used only one finishing and polishing methodology, which could have affected the surface properties of chemically different materials. Because this is an in vitro study and the materials have intrinsic manufacturing faults, handling the materials during glazing, polishing, and finishing could have influenced the outcome. Because of the aforementioned constraints, it is therefore important to evaluate the current study’s results cautiously. To better understand the surface characteristics of these cutting-edge indirect CAD/CAM restorative materials with various factors, and to overcome the limitations of this study, more research is needed.

## 5. Conclusions

Within the limitations of this study, we found the following:

- significant variations in the surface roughness (Ra) were found between the finished/polished and glazed surfaces of the five test materials;
- hybrid ceramics exhibited the highest Ra values for the finished/polished surfaces as compared to other test materials;
- zirconia exhibited the highest Ra values among the glazed surfaces of the test materials;
- the Ra values of both the finished/polished or glazed surfaces of the tested materials were within the clinically acceptable range (0.2–0.5 μm), except for the glazed surface of the zirconia ceramics (0.84 μm).

## Figures and Tables

**Table 1:** Details of the indirect CAD/CAM materials to be tested in the study (N = 75).

Test Material	Abbreviation	Brand	Manufacturer	Composition	LOT Number
Composite	TC	Tetric^®^ CAD	Ivoclar Vivadent, Schaan, Lichtenstein	64% Barium glass, 7.1% SiO_2_, 28.4% Dimethacrylates and 0.5% Pigments	Y28816
Lithium-Disilicate-Glass-ceramics	LS_2_	IPS e.max^®^ CAD	Ivoclar Vivadent, Schaan, Lichtenstein	5.0–11.0% P_2_O_5_, 0.0–8.0% ZnO, 0.0–5.0% Al_2_O_3_, 0.0–5.0% MgO, 11.0–19.0% Li_2_O, 0.0–13.0% K_2_O, 57.0–80.0% SiO_2_, 0.0–8.0% ZrO_2_ Coloring Oxides	Z02JYZ
Zirconium Oxide ceramics	ZrO_2_	IPS e.max^®^ ZirCAD	Ivoclar Vivadent, Schaan, Lichtenstein	88.0–95.5% ZrO_2_, >4.5%–≤7.0% Y_2_O_3_, ≤5.0% HfO_2_, ≤1.0% Al_2_O_3_, ≤1.5%; Other oxides	X16329
Zr-reinforced, Lithium Silicate	ZLS	CELTRA^®^ Duo	Dentsply-Sirona, Bensheim, Germany	10% ZrO_2_, 90% Lithium Disilicate	16010732
Hybrid-Ceramic	HC	Vita Enamic^®^	VITA, Zahnfabrik, Germany	86% Ceramic, 14% Polymer	98520

Silicon dioxide (SiO_2_); lithium oxide (Li_2_O); potassium oxide (K_2_O); zirconium dioxide (ZrO_2_); zinc oxide (ZnO); aluminum oxide (Al_2_O_3_); magnesium oxide (MgO), yttrium oxide (Y_2_O_3_); hafnium oxide (HfO_2_).

**Table 2:** Descriptive statistics of surface roughness (Ra) for the two methods of surface conditioning (glazed and finished/polished) for each of the five research materials.

Type of Material	Surface Conditioning			
	Finished/Polished (μm)		Glazed (μm)	
	Mean (Sd.,)	Median (IQR)	Mean (Sd.,)	Median (IQR)
Tetric CAD	0.1801 (0.029)	0.1887 (0.026)	0.0860 (0.054)	0.0740 (0.085)
IPS e.max CAD	0.1922 (0.024)	0.1930 (0.037)	0.1191 (0.031)	0.1223 (0.063)
IPS e.max ZirCAD	0.2910 (0.082)	0.3090 (0.140)	0.8493 (0.604)	0.4897 (1.064)
Celtra Due	0.1828 (0.024)	0.1930 (0.041)	0.0830 (0.083)	0.0573 (0.020)
Vita Enamic	0.4644 (0.137)	0.4343 (0.202)	0.0937 (0.050)	0.0813 (0.062)

**Table 3:** Comparison of mean ranks of surface roughness (Ra) among the five study materials for the finished/polished surfaces by post hoc Conover test.

Type of Material	Mean Ranks (μm)	*p*-Value
Tetric CAD	21.60	<0.0001
IPS e.max CAD	27.47	
IPS e.max ZirCAD	50.33 *	
Celtra Due	24.40	
Vita Enamic	66.20 **	

* Significantly higher than Celtra Due, IPS e.max CAD, and Tetric CAD and significantly lower than Vita Enamic. ** Significantly higher than Celtra Due, IPS e.max CAD, Tetric CAD, and Vita Enamic.

**Table 4:** Comparison of mean ranks of surface roughness (Ra) among the five study materials for the glazed surface by post hoc Conover test.

Type of Material	Mean Ranks (μm)	*p*-Value
Tetric CAD	27.87	<0.0001
IPS e.max CAD	41.63 *	
IPS e.max ZirCAD	67.73 **	
Celtra Due	21.53	
Vita Enamic	31.23	

* Significantly higher than Celtra Due and Tetric CAD and significantly lower than IPS e.max ZirCAD. ** Significantly higher than Celtra Due, IPS e.max CAD, Tetric CAD, IPS e.max ZirCAD, and Vita Enamic.

**Table 5:** Comparison of mean ranks of surface roughness (Ra) for the two types of surface conditioning within each of five study materials with Mann–Whitney U-test.

Type of Material	Type of Treatment		*p*-Value
	Finished/Polished (μm)	Glazed (μm)	
Tetric CAD	21.97	9.03	<0.0001
IPS e.max CAD	23.00	8.00	<0.0001
IPS e.max ZirCAD	9.40	21.60	<0.0001
Celtra Due	22.00	9.00	<0.0001
Vita Enamic	23.00	8.00	<0.0001

## Data Availability

Data are available on request from corresponding author due to ethical reasons.
